# Post market pilot programme with single pulse transcranial magnetic stimulation (sTMS) for acute treatment of migraine: SpringTMS™ use in migraine

**DOI:** 10.1186/1129-2377-1-S14-P222

**Published:** 2013-02-21

**Authors:** MW Weatherall, R Bhola, N Giffin, PJ Goadsby

**Affiliations:** 1 Princess Margaret Migraine Clinic, London, UK; 2 eNeura Therapeutics, California, USA; 3 Royal United Hospital, Bath, UK; 4 UCSF, San Francisco, USA

## Introduction

Some patients suffer disabling, frequent migraine without effective treatment as current pharmacological options are either contra-indicated, poorly tolerated or overused. A post market pilot programme with the sTMS device was initiated for patients with migraine.

## Aims and objectives

To evaluate patient response to sTMS in an open outpatient setting - To assess the impact of treating migraine attacks with single pulse sTMS on pain and associated migraine symptoms over an extended period (minimum three months) - Understand patient support and educational needs for using sTMS - Assist patients in establishing an optimal sTMS treatment scheme for their migraine patterns - Review options for sTMS within the UK headache care pathway.

## Methods

Clinicians selected patients and prescribed the device. Patients subsequently received the device to use for a minimum period of three months. A clinical liaison had first contact with the patient to discuss treatment and use. Monthly telephone reviews were conducted to support and monitor the patients’ treatment and progress. Survey data was collected monthly over the treatment period. The patients’ progress with treatment was reported to their clinician.

## Results

Sixty-one patients have been prescribed sTMS from which 37 (61%) have been using the device for a minimum of three months and completed surveys. A reduction or alleviation of pain was reported by 73%. Associated symptoms were improved in 63% of patients or for some, did not develop. A reduction in the number of headache days was reported by 53%. When using the combination of sTMS and a medicine, 30% reported no headache recurrence. Quality of sleep improved in 17%. The treatment was well tolerated with no adverse events reported. Conclusion: The sTMS device is a new and effective acute migraine treatment. This CE marked device is safe to use in clinical practice and has reliable, reproducible effects on migraine over time.

